# EDCs Mixtures: A Stealthy Hazard for Human Health?

**DOI:** 10.3390/toxics5010005

**Published:** 2017-02-07

**Authors:** Edna Ribeiro, Carina Ladeira, Susana Viegas

**Affiliations:** 1 Environment and Health Research Group, Lisbon School of Health Technology, Polytechnic Institute of Lisbon (ESTeSL/IPL), Av. D. João II, lote 4.69.01, 1990-096 Parque das Nações, Lisboa, Portugal; carina.ladeira@estesl.ipl.pt (C.L.); susana.viegas@estesl.ipl.pt (S.V.); 2 Landscape, Environment, Agriculture and Food (LEAF), Instituto Superior de Agronomia, Universidade de Lisboa, Tapada da Ajuda, 1349-017 Lisboa, Portugal; 3 Research Group in Genetics and Metabolism (GIGM), Lisbon School of Health Technology, Polytechnic Institute of Lisbon (ESTeSL/IPL), Av. D. João II, lote 4.69.01, 1990-096 Parque das Nações, Lisboa, Portugal; 4 Centro de Investigação e Estudos em Saúde Pública, Escola Nacional de Saúde Pública, ENSP, Universidade Nova de Lisboa, 1900 Lisbon, Portugal

**Keywords:** endocrine disrupting chemicals (EDCs), mixtures, biomonitoring, windows of exposure, risk assessment

## Abstract

Endocrine disrupting chemicals (EDCs) are exogenous chemicals that may occur naturally (e.g., phytoestrogens), while others are industrial substances and plasticizers commonly utilized worldwide to which human exposure, particularly at low-doses, is omnipresent, persistent and occurs in complex mixtures. EDCs can interfere with/or mimic estrogenic hormones and, consequently, can simultaneously trigger diverse signaling pathways which result in diverse and divergent biological responses. Additionally, EDCs can also bioaccumulate in lipid compartments of the organism forming a mixed “body burden” of contaminants. Although the independent action of chemicals has been considered the main principle in EDCs mixture toxicity, recent studies have demonstrated that numerous effects cannot be predicted when analyzing single compounds independently. Co-exposure to these agents, particularly in critical windows of exposure, may induce hazardous health effects potentially associated with a complex “body burden” of different origins. Here, we performed an exhaustive review of the available literature regarding EDCs mixtures exposure, toxicity mechanisms and effects, particularly at the most vulnerable human life stages. Although the assessment of potential risks to human health due to exposure to EDCs mixtures is a major topic for consumer safety, information regarding effective mixtures effects is still scarce.

## 1. Introduction

Endocrine disruption associate to environmental exposures has been acknowledged for several years. During the last decades, endocrine disruption research has been directed to the reproductive system, since an increase of reproductive system health problems was demonstrated. Additionally, the increment of hormone-related cancers (e.g., testicular and breast cancers), decreased semen quality, and incidence of birth anomalies in genital organs may also be correlated to environmental factors [1]. Our concern with human exposure to Endocrine Disrupting Chemicals (EDCs) derives from: (i) evidence regarding molecular mechanisms correlated to effects at very low doses; (ii) in vitro effects in animal models resultant from doses within the range of human exposure; and (iii) extensive and prevalent human exposure to EDCs at concentrations that have been documented to endorse adverse effects in animals [2]. The inadvertent exposure of humans to numerous chemicals through divergent routes establishes a “mixture” context that is the focus of our review paper. Similar to the most recent trends explained in Evans et al. [3], we do not discuss commercial products with defined ingredients, characterized as “intentional” mixtures. A mixture does not directly imply a risk to human or environmental health per se, however existing experimental data regarding mixture effects of low and/or ineffective levels highlight the urge to implement valuable and effective assessments with the ability to evaluate whether more accurate estimations of risk should be produced by considering the existent chemicals [4,5,6]. It is currently acknowledged that humans are exposed to diverse chemicals through several exposure routes, which can be measured in human biological samples, including blood and breast milk. However, the potential risk to human health associated to the acquired “body burden” of contaminants is still not assessed or managed by regulatory organizations, or even routinely monitored. 

## 2. EDCs State of the Art

Endocrine disruptors or endocrine disrupting chemicals are omnipresent exogenous compounds with the ability to behave as biological signals and as a result can interfere with/or mimic endogenous estrogenic hormones, acting as agonists or antagonists [7]. The U.S. Environmental Protection Agency (EPA) classifies environmental EDCs as “an exogenous agent that interferes with production, release, transport, metabolism, binding, action, or elimination of natural hormones in the body responsible for the maintenance of homeostasis and the regulation of developmental processes”. Furthermore, the International Programme on Chemical Safety (IPCS) defined possible EDC from actual EDC, as “an exogenous substance or mixture that possesses properties that might be expected to lead to endocrine disruption in intact organisms, or in progeny, or (sub) populations” [8]. Exposure to these compounds is accountable for different and divergent biological responses associated with the simultaneous and differential activation of specific signaling pathways in diverse cell types [9]. Although around 800 commercial substances are supposed to affect the endocrine system, only a minor portion of these have been tested for possible hazardous effects [10]. Human exposure, particularly at extremely low-doses, is generalized, persistent and occurs in different mixtures with potential associated effects that may not be predictable when evaluating single compounds per se [11]. Therefore, the assessment of potential risks to human health resultant of exposure to EDCs and mixtures is a key and foremost important subject for consumer safety. EDCs may occur naturally (e.g., phytoestrogens), whereas others are commercial substances and plasticizers globally utilized in the plastic industry. EDCs comprise persistent organic pollutants (POPs) (e.g., polychlorinated biphenyls (PCBs)), polychlorinated dibenzo-p-dioxins/furans (PCDDs/PCDFs), organochlorine pesticides (OCPs) and polybrominated flame-retardants [12,13]. Diet, particularly ingestion of contaminated food, is estimated to be the main source of human exposure to EDCs for all age groups [14]. It is important to note that dietary habits are influenced by diverse factors such as socioeconomic status, culture and religion as well as individual choices. These factors have a major impact on human daily consumption of nutrients, bioactive constituents, residues and contaminants. Additionally, some foods of vegetable origin are a source of natural endocrine-active substances (e.g., phytoestrogens) that can interact with endogenous estrogen signaling pathways [12]. Currently, the worldwide spread of vegetarianism has led to the increased intake of vegetables such as soybeans with a high content of genistein, one of the most studied isoflavones associated with several biological activities, including as a phytoestrogen, an antioxidant, and as an inhibitor of a broad range of tyrosine kinases [15,16,17].

Considering human exposure patterns through oral intake EDCs, can be separated in four major categories:

− EDCs with bioaccumulation ability (e.g., polychlorinated biphenyls -PCBs-, polybrominated flame retardants, perfluorinated chemicals);
− Compounds utilized in food production (e.g., pesticides);
− Chemicals present in food due to contact materials, processing aids, etc. (e.g., BPA); and
− Endocrine-active substances naturally present in food (e.g., genistein).

Another significant source of human exposure to EDCs, besides dietary route, is the indoor environment. Indoor air contamination in buildings and houses may induce airborne exposure noticeably above background levels. One group of the relevant compounds associated with indoor exposures are PCBs [18]. Although PCBs were banned in several countries due to indication of environmental accumulation and potential hazardous health effects, these compound are currently present in the environment, indoors and humans and are classified as persistent organic pollutants [18]. Exposures to PCBs have been positively correlated with adverse liver, kidney, endocrine, and neurodevelopmental effects, and thyroid disruption has also been reported [19]. However, PCBs effects on humans associated with acute exposures or long-term effects on reproduction are still a matter of debate in the scientific community. Currently, the project HESPERUS is the first study that tends to evaluate potential hazardous health effects of continuous exposure to the lower-chlorinated, semi-volatile PCBs in indoor environments [18]. Thus, considering that EDCs are also detected in waste water, drinking water, air and dust particles from both house dust samples and urban ambient outdoor environment [20,21,22], non-dietary human exposure sources such as air or contact must not be underestimated [23,24].

Furthermore, it is important to note that hormones, as well as EDCs responses, do not follow the classical monotonic dose responses typically used in toxicological risk assessments but non-monotonic dose responses (NMDR) curves which can result from multiple mechanisms [2,25]. The “low-dose” effect was defined based on the Environmental Protection Agency (USEPA) criterion for low-dose effects of EDCs as those observed at concentrations below the levels used for classical toxicological assessments [26]. Remarkably, extremely low levels are able to stimulate receptor up-regulation, resulting in response enhancement, whereas higher doses (within the classic toxicological range) can induce receptor down-regulation, resulting in a diminished responses [27]. In single response experiments, NMDR curves are a common finding for EDCs. An additional difficulty, however, is correlated to the assessment of multiple outcomes as qualitatively divergent outcomes are frequently observed at low and high EDCs exposures. One hypothesis for this phenomenon is that the patterns of genes whose expression is affected by low doses of endogenous hormones may be divergent from the set of genes altered by high concentrations. In increasing concentrations, hormones and hormone-mimicking chemicals may signal different receptors, defined as receptor cross-talk [27]. In the case of the xenoestrogen BPA, NMDR have been demonstrated to emerge on exposed pituitary, prostate and pancreatic cultured cells, since very low doses can induce significant effects that are not detectable at higher concentrations (reviewed in [25]). Furthermore, EDCs effects can also be mediated by mechanisms external to direct mediation by classical hormone receptors. Nonspecific (non-receptor-mediated) effects may occur exclusively at high doses. Moreover, EDCs can also interact with the synthesis or function of enzymes responsible for the synthesis or degradation of hormones as well as of coregulatory proteins which interact with receptors and, in the situation of neurologic actions, alter neurotransmitters and associated receptors [27]. Moreover, it is imperative to consider that individuals environmentally exposed to EDCs may also be occupationally exposed, to the same and/or different compounds which leads to the notion that the evaluation of potential aggregate exposures is crucial for the development of an effective risk assessments.

## 3. Materials and Methods

An exhaustive search was performed for papers available in scientific databases reporting EDCs mixtures effects and associated mechanisms. The articles presented and discussed were obtained using diverse scientific databases such as PubMed and Google Scholar and using the keywords: Endocrine disrupting chemicals (EDCs); EDCs Mixtures; Mixture Effects; Toxicity Mechanisms; Biomonitoring; Risk Assessment and Critical Windows of Exposure. In our review, we considered experimental studies, epidemiological studies and previous reviews published in 2000–2016. Inclusion and exclusion criteria were established before bibliographic search. Studies published in languages other than English were considered only if an abstract was available. The articles that, besides written in English, also reported findings regarding EDCs mixtures exposure, toxicity mechanisms and effects in vivo and in vitro, biomonitoring in pregnant women, early life effects and reports of male vulnerability, were chosen for further analysis. Regarding in vitro EDCs mixture effects in human cell lines, only 14 studies were considered, which are summarized in Table 1.

## 4. EDCs Mixtures Toxicity Mechanisms

Chemical regulation operates almost entirely on a chemical-by-chemical basis, however the concern of whether this approach is suitably protective if different chemicals have the same toxic effect remains. Humans are unquestionably exposed to numerous chemicals at a time, found in food, air, drinking water, household, consumer products, and cosmetics [3]. Mixture risk assessment (MRA) defines the assessment of the cumulative risk to human health or the environment due to exposure to different chemicals via multiple routes [3]. The toxicological effects of compounds mixtures can result in either independence (response additivity) or dose addition. Concentration (or dose) addition has been used in association to mixtures of similar acting chemicals such as those interacting with ER, while independent action has been employed for different acting compounds, including compounds in a mixture that interact with dissimilar receptors or other molecular targets [28]. In 2011, the European Centre for Ecotoxicology and Toxicology of Chemicals (ECETOC) accomplished an extensive assessment of compounds mixture studies tested at concentrations in the range of the No Observed Adverse Effect Level (NOAEL) or No Observed Effect Concentration (NOEC) or below for each constituent in the mixture. As a result, the independent action of chemicals was considered the main principle of mixture toxicity and the report stated that “there is no evidence that exposure to complex mixtures of components, each well-regulated according to established risk assessment approaches, would pose a health risk to humans” [29]. As similarly acting chemicals can substitute one another, without decreased efficiency, combination effects can be predictable, even at doses below NOAELs. Thus, classical risk assessment approaches focused on single chemicals assessments are problematic, since considerations of individual NOAEL do not integrate potential risks without information regarding simultaneous exposure to other occurring agents [6,30]. Additionally, the World Health Organization (WHO) report on endocrine disrupting chemicals also specified: “…there is emerging evidence that many chemicals may act additively and, each at levels without individual effect, could act together to cause health problems” [10]. EFSA considered that compounds with equal effects in the same target organ are presumed to act in an additive way, even when their chemical structures and molecular mechanisms are unlike (reviewed in [13]). The situation is thought to be entirely different when exposure occurs with substances that have different modes of action. Because these chemicals interact individually with different subsystems of the affected organisms, the supposition is that, as long as the concentration of each constituent stays below its NOAEL, mixtures pose no valuable health concerns [6]. However, some EDCs (e.g., BPA) can bind to numerous hormone receptors. Similarly, the concept of threshold as it applies to EDCs is complex and must be evaluated with caution [31]. Studies focused in evaluation of mixtures of components with estrogenic, antiandrogenic, and thyroid-disrupting activities demonstrated the utility of the concept of dose addition in expecting mixture effects, demonstrating that joint effects occur even at levels below doses that induce observable effects (reviewed in [4]). Dose additivity has been rationalized, particularly in the case of compounds competing independently for the same receptor [32,33]. In human breast carcinoma MVLN cells and Chinese hamster ovary CHO-K1 cells, mixtures of currently used pesticides, namely, bitertanol, propiconazole, cypermethrin, terbuthylazine, bitertanol, propiconazole, cypermethrin, and malathion, demonstrated endocrine-disrupting potential in vitro, potentially mediated via ER, AR and aromatase activities [34]. In multipotent murine mesenchymal stem cells (C3H10T1/2), simultaneous exposure of BPA, diethylhexylphthalate (DEHP) and tributyltin (TBT), known to interfere with adipogenesis, increased the development of adipocytes and the expression of adipogenic marker genes [35]. Co-exposure to BPA and nonylphenol (NP) endorsed an additive effect on some antioxidant parameters in zebrafish embryos [36]. In addition, a recent yeast estrogen assay (YES) that evaluated the estrogenic potential of mixtures of genistein, BPA and/or trenbolone, which exert their estrogenic effects through analogous mechanisms (i.e., binding to estrogen receptor) demonstrated that the dose additivity of estrogenic mixtures of genistein, BPA and/or trenbolone is established by receptor occupancy [37]. Additionally, a recent study has also demonstrated that combined exposures to BPA and soy-based phytoestrogens results in additive estrogenic effects which can play key roles in the etiology of estrogen-linked diseases, such as breast cancer [38]. Conversely, other studies have demonstrated that dose additivity of estrogenic mixtures is not the only mechanism of mixture toxicity, which leads to a controversy regarding the effective effects of these mixtures for human health. In ovarian cancer cell line BG-1, genistein, was reported to efficiently suppress BPA-induced ERα mediated proliferation through inhibition of cell cycle progression [39]. Additionally, besides the fact that BPA can promote estrogen-like effects similar to, or stronger than, E2 [40], it is also capable to interact with this endogenous hormone. In MCF-7 breast cancer cell line, combined exposure to BPA at low dose and E2 at physiological concentration induces cell proliferation and decreases apoptosis [41]. In addition, in colon cancer cell line DLD-1 E2 pro-apoptotic action is antagonized by BPA through inhibition of cascade 3 activation [42]. A recent study performed using a goldfish model demonstrated that mixture exposure effects are characterized by a stress response that cannot be predicted from exposure to individual compounds, even in the absence of further phenotypic features [43]. In addition, synergistic effects were observed for reactive oxidative species (ROS) induction in human hepatocarcinoma (HepG2) cells exposed to perfluorinated and brominated mixture in contrast to the effects of the individual mixtures [44]. Additionally, in male Japanese medaka, the assessment of EDCs mixtures in hypothalamic–pituitary–gonadal axis gene transcription also demonstrated that, although exposure to flutamide and vinclozolin per se resulted in similar transcriptional responses, mixture effects could not be extrapolated based on the results from single compounds [45].

Moreover, considering that many EDCs bioaccumulate in lipid compartments of organisms forming a mixed “body burden” of contaminants of diverse origins, it is assured that the daily dose of each compound alone is not the only one that should be taken into consideration [13]. Thus, mixture effects of EDCs certainly extend considerably beyond the groups of chemicals with similar chemical structures and molecular mechanisms, as summarized in Table 1 by in vitro studies that assessed EDCs mixtures effects in human cell lines.

The debate on whether risks associated with low-level exposure to different chemicals can be assessed without considering the mode of action of the chemicals present in the “cocktail of pollutants” is still intense. In view of the variety of “real world” mixtures composed by several chemicals with a multitude of dissimilar modes of action, divergent action of mixture constituents should be considered as the default scenario [4]. Considering the limitations of the quantity of chemicals and animals that can be manageable in laboratory experiments and the complexity of the assessment of effective human in vivo concentrations, mixture effects at “realistic” levels are extremely difficult to evaluate. However this may not present a definitive obstacle to risk assessment. The understanding of the determinants of additive effects is now satisfactorily advanced to allow anticipation of mixture effects through mathematical modeling. Difficulties present themselves, however, when appropriate effect data for single EDCs are unavailable. In these cases, mixture effects cannot be predicted [6].

## 5. Critical Windows of Exposure

EDCs exposures health risks are closely associated to particular stages of life with concomitant critical windows of exposure [13]. Considering EDCs mechanisms of action, embryonic development and early life stages are certainly key critical windows of human exposure to these compounds. Early development requires accurate timing of hormone action to endorse proper tissues and organs growth and development. EDCs can affect the endogenous functioning of these hormones and with enzymes implicated in xenobiotic biotransformation. Additionally, it is imperative to consider that, at these stages, elimination processes are not fully developed [46,47]. Thus, during prenatal and early life stages of human development, exposing fetuses and young children to pollutants, such as EDCs, through maternal blood and/or milk may enduringly reprogram physiologic processes prompting health and/or reproductive dysfunctions later in life [48].

### 5.1. Embryonic Development

Exposure effects to environmental chemicals, which can comprise some EDCs, include direct alterations on physiological mechanisms associated with gonadal development and function [49]. Pregnant women are exposed to mixtures of EDCs during pregnancy. Separating potential effects of EDC exposure per se is extremely challenging, particularly when exposures are associated to common sources. For example, serum levels of individual polychlorinated biphenyl (PCB) congeners are linked with one another as well as with some organochlorine (OC) pesticides [50]. Additionally, numerous studies have proven the transplacental transfer of xenoestrogens including POPs, flame retardants and arsenic [51,52,53]. In addition, considerably high concentrations of BPA have been measured in human placental samples as well as in fetal serum indicating that the placenta does not work as a barrier to BPA and therefore developing human fetuses are chronically exposed to BPA with higher levels detected in male fetuses [54]. Furthermore, glucuronidase-mediated BPA deconjugation in placenta and fetal tissues has been advocated to contribute to substantial fetus exposure to free bioactive BPA [55]. Although estimating human exposure to BPA is still a controversial issue and several inconsistencies have been described between the estimate intake levels and biomonitoring studies performed in human blood/serum samples [22], BPA pharmacokinetic models based on hasty BPA glucuronidation and urinary clearance [56,57] do not take into consideration possible BPA deconjugation processes in specific tissues such as placenta. During early embryonic development, probable EDCs targets comprise cell cleavage and differentiation, cell lineage determination, methylation, implantation, maintenance of pregnancy and organogenesis [49]. A recently published study demonstrated that in utero exposure to xenoestrogens may modify the placenta epigenome and that male descendants appear to be particularly vulnerable to xenoestrogens exposure during prenatal development, associated with alterations in DNA methylation of specific genomic repetitive sequences [58]. Additionally, some suggestive genes formerly correlated to birth weight, Type 2 diabetes, obesity or steroid hormone signaling were found to be differentially methylated in males as a result of prenatal xenoestrogen exposure [59]. Despise the scarce information regarding BPA effects on occupationally exposed individuals, exposed to higher BPA levels, epidemiological studies demonstrated that parental occupational exposure to this compound during pregnancy is correlated with decreased birth weight of offspring [60] and with shortened anogenital distance in male offspring [61]. Moreover, a recently published study demonstrated that perinatal exposure to EDCs mixtures relevant in the context of human exposure resulted in altered female rat reproductive parameters such as reduced follicle in pre-pubertal animals and relevantly in adult animals evidences for early female reproductive senescence have also been described [62]. In addition, in zebrafish model, EDCs exposure of individual compounds as well as their mixtures results in sexual differentiation effects associated to histopathological alterations in the gonads of both males and females, with a sex ratio tendency towards females and to permanent disruption of sexual development [63]. Understanding developmental exposure effects of mixtures of EDCs and its underling mechanisms is critical to public health protection, particularly in the context of both male and female reproductive disorders.

### 5.2. Early Life

The Developmental Origins of Health and Disease (DOHaD) hypothesis advocates that early life experiences can have a significant impact in health outcomes later in life [64]. Newborns are exposed to EDCs beginning during embryonic development, as discussed above, and therefore an early body burden is probably derivative from in utero exposure. Cumulated evidence has emphasized the critical role of early life environment in determining health outcomes of individuals and increasing recent studies have demonstrated that early life perturbations affect the health of the succeeding generations [65]. For small infants, breast milk and artificial milk are the main sources of exposure. Mixtures of lipophilic compounds (PCBs, brominated flame retardants, and dioxins) can be transferred from the parental body burden to the newborn through breastfeeding, as breast-fed infants are a population group with higher PBDE intake [66]. In the case of BPA, the most recent worldwide biomonitoring study based on data from urinary BPA concentrations estimated human exposure to be 0.27 μg/kg body weight/day for the general population, 0.78 μg/kg body weight/day for children and 0.45–1.61 μg/kg body weight/day for infants [67]. In addition to the contamination of breast milk through the maternal exposure, the contamination of items utilized for nursing can also determine the safety of newborns and infants diet. Although the various scientific assessments conducted by the EFSA have recurrently concluded that there is no concern for human health [68], in 2011 the European Legislation banned the use of BPA in the manufacture of baby bottles [69]. Another point of debate is the immaturity of both hepatic detoxification and blood–brain barrier, which results in limited internal defenses of neonates and infants against contaminants [70]. Considering the current pharmacokinetic models, these factors increase the bioavailability of compounds such as BPA that undertake extensive gut and liver glucuronidation, which leads to decreased levels of unmetabolized bioactive BPA that enters the blood stream [56].

## 6. Male Vulnerability to EDCs

Several studies that focus on EDCs effects during embryonic development and early life, as discussed above, indicate that, although both genders are affected by exposure to EDCs mixtures, males appear to be particularly vulnerable to exposure of certain compounds. The epidemic increase of male reproductive problems, which cannot be explained by genetic changes, has occurred contemporaneously with cumulative exposures to various environmental factors through modern lifestyle. Increasing evidence demonstrates that such exposures include EDCs mixtures which may be associated with male reproductive disorders and diseases [71]. There has been substantial controversy regarding the capacity of EDCs at lower than threshold concentrations levels to act together, particularly when they have different mechanisms of action. Recent studies have demonstrated that low dose mixtures of chemical reducing androgens, through different mechanisms, resulted in additive male reproductive dysfunction [72,73]. Moreover, the antiandrogenic action of six binaries and one ternary mixture of phthalates displaying complete antiandrogenic dose–response curves, and additionally binary mixtures of phthalates and BPA at equi-effective doses evaluated in MDA-kb2 cells, displayed a concentration addition model with a propensity to synergism at higher and antagonism at lower doses [74]. Binary mixtures of five typical estrogens (estrone (E1), 17β-estradiol (E2), 4-n-octylphenol (4-n-OP), 4-n-nonylphenol (4-n-NP) and BPA) resulted in an additive effect on serum vitellogenin increase in male goldfish at low doses, and divergences associated with high dose exposures with predicted additive effects exceeding those observed. Severe gonads atrophy was also described in all the mixture treatment groups [75]. Exposure to mixtures of atrazine (ATR), perfluorooctanoic acid (PFOA), BPA and 2,3,7,8-tetrachlorodibenzo-p-dioxin (TCDD) in pregnant mice resulted in significant effects such as reversed lower neophobia levels in male offspring, showing a potential of these mixtures for enhanced behavioral effects in males [76]. In occupational exposure context, increased mean serum of BPA concentration has been associated with diminished mean androstenedione (AD) level (0.18 ng/mL, 95% CI −0.22 to −0.13) and increased mean serum sex hormone-binding globulin (SHBG) level (2.79 nmol/L, 95% CI 2.11–3.46) in male workers [77]. Additionally, BPA has also been positively correlated with decreased semen quality and male infertility, for which induction of germ cell apoptosis is considered a primary contributing factor [78], reduced sperm concentration, total sperm count, vitality and motility [79], and alterations in laboratory parameters that may contribute to male infertility such as, prolactin, estradiol and sex hormone-binding globulin (SHBG) [80]. Thus, the existing studies indicate that BPA-exposed male workers had consistently higher risk of sexual dysfunction among all domains of male sexual function than unexposed workers [81]. Additionally, PCB exposure has also been associated with increased anogenital distance and prostate size and with diminished epididymal weight, epididymal sperm count, and motile epididymal sperm count (reviewed in [82]). Furthermore, in vitro experiments have also demonstrated that low concentrations of the phytoestrogen genistein, nonylphenol and 8-pre-nylnaringenin are more effective when combined in relation to the effects of the compounds per se, on key processes of capacitation and acrosome reaction in human and mouse spermatozoa [12]. Alterations in male reproductive functions, in rat, were reported to be more marked in co-exposure experiments to genistein at low-dose (1 mg/kg/day) and the fungicide vinclozolin (1 mg/kg/day) from conception to adulthood. On the other hand, similar to BPA, maternal genistein supplement can decrease body weight in male offspring [83] and decreased percentage of normal sperm and augmented abnormalities in semen morphology [84]. Additionally, genistein levels were reported to be 2.67 times higher in patients with precocious puberty than those of the control which indicate a potential role on this condition [85]. Concurrent with accumulating evidence from studies suggesting that EDCs may contribute to male reproductive disorders and diseases, the burden of male reproductive health problems is significant not only at individual and population levels but also in economical contexts. Prevention of EDCs exposures has the potential to reduce the incidence of several male reproductive disorders and diseases, and their associated health care and other social costs, which, in the European Union, totals approximately €15 billion annually [71].

## 7. In Vitro Mixtures Effects

In vitro cell systems have been widely utilized for the assessment of low-dose effects of well-known EDCs per se, and most studies have been directed to human cancer cell lines, particularly breast cancer with associated effects on proliferation and gene expression (reviewed in [86]). After article selection process, 14 articles were considered important for this review of EDCs mixtures effects in human cell lines. Table 1 summarizes the reported effects of several EDCs mixtures in the studied concentrations and utilized cell lines. In accordance with preceding data, our work also demonstrated that breast cancer cells, particularly MCF7, are the most utilized cell lines for the assessment of EDCs mixtures effects, although other cell lines have also been evaluated. Interestingly, in MCF7, divergent results were reported when cells were exposed to different EDCs mixtures including additive, greater-than-additive, antagonistic and synergistic interactions. Considering that activation of divergent signaling pathways has been described for exposure to different concentrations of single EDCs [87], we can postulate that, according to the reported findings, the same cell and tissues can respond differently to EDCs mixtures, potentially by activation of different signaling pathways.

The term “low-dose” effects defines biological changes occurring in the concentration range of typical human exposures or lower than the expected NOAEL level used to establish the oral reference dosage (RfD) [88]. For the past years, “low-dose” effects have been a focal point of numerous studies (reviewed in [89]). Here, we report that most of the performed studies have been conducted using EDCs “low dose” mixture concentrations in the range of 10 pM to 10 µM, whereas a single study evaluated both low and high doses effects of NP (0–100 μM) and BPA (0–5000 μM) [83]. Interestingly, the most evaluated EDC in mixtures was BPA in the range of “low doses” [90]. 

Regarding EDCs mixture effects, the most frequently observed were undoubtedly additive effects, particularly associated with cellular proliferation [38,90,91,92,93,94,95]. Nevertheless, greater-than-additive interaction effects were also observed [89,93,95] as well as antagonistic and synergistic effects in cell proliferation [90,92,94,96]. EDCs mixtures demonstrated endocrine disrupting potential via both Estrogen (ER) and Androgen (AR) pathways [34]. Among the ER-dependent effects, we identified additive interactions on estrogen receptor activation and estrogen-regulated pS2 gene transcription in MCF7 cells [97], in Ovarian cancer cell line BG-1 suppression of BPA Estrogen receptor alpha (ERα) mediated cell proliferation induced by genistein [39] and ER α and ER β activation additive effects in MCF7 and HeLa cells [38]. Other EDCs mixture effects such as enhanced Ca2+ response [98], reduction of phagocytosis disturbance of TNF-α, IL-1 β and IL-8 cytokine secretions [99] and synergistic effects in ROS induction [44] were also identified.

The results collected here clearly demonstrate that dose additivity, which has been utilized to characterize mixtures effects of similar acting compounds such as EDCs [28] is not the only mechanism of mixture toxicity and that in the same in vitro cell system (MCF7) exposure to different EDCs mixtures results in divergent effects (e.g., Additive, Antagonistic and Synergistic).

## 8. Discussion

EDCs account for a substantial amount of chemicals that humans are constantly and continuously environmentally exposed. Many of these chemicals are a result of industrialization and technological development in the past century [7]. The current media scrutiny regarding the presence of harmful chemicals in food and consumer goods has led to general public awareness of the effective simultaneous exposure to multiple chemicals during daily life. Although the urge to assess combined exposure effects is growing among experts, risk assessment of combined exposures such as EDCs mixtures still faces major challenges [4], and potential hazardous effects of EDCs are still speculated based in findings from classical epidemiological data of individuals and population studies [100]. There is irrefutable evidence across multiple chemicals and organ systems concerning EDCs adverse effects in humans and human cells, however the doses at which these effects may occur (occupational vs. environmental exposures) remain unresolved for several compounds [31] and chemicals risk assessment, particularly in the context of health risk assessment, typically does not take in account effects of combined exposures. Additionally, EDCs, similar to hormones, do follow non-monotonic dose responses (NMDR) curves such as inverted U-shaped curves, which means that low dose exposures effects cannot be predicted by observed effects of higher doses, which increases the difficulty for risk assessment and the potential of mixtures unpredictable interactions [100].

Furthermore, the complexity of EDCs mixtures concerning compounds with short or long half-lives is also of key importance to conduct valuable risk assessments. It is acknowledged that compounds with long half-lives have higher reliability in exposure assessments, whereas short-half-life EDCs are rapidly metabolized and eliminated [100]. However, these claims do not take into account compounds that can be bioaccumulated in particular tissues of the human organism. In the case of BPA, considered a short-half-life EDCs [56,57], several inconsistencies have been described between estimated intake levels obtained from urinary human samples and biomonitoring studies performed in human blood/serum regarding the steady-state presence of unmetabolized BPA [22], which can be 10-fold higher than the worst case predictions for daily exposure [101]. In addition, substantially high concentrations of BPA measured in human placental samples when compared to urine or blood/serum indicates a potential for bioaccumulation. It is currently acknowledged that health hazardous outcomes associated with EDCs exposures may be closely associated with particular vulnerable life stages with concomitant critical windows of exposure [13]. Embryonic development and early life stages are most certainly critical windows of exposure to EDCs, as early development requires accurate timing of hormone action to promote proper tissues and organs growth and development.

During pregnancy, mother and embryo/fetus establish an extremely intimate contact in which several molecules are involved. In the feto–maternal interface, hormones such as estrogens and progesterone are major players for the necessary and accurate endocrine regulation in which the placenta has a central role. Human placenta is crucial to sustain pregnancy and assure fetal development and growth, and it is assumed to be a protective organ capable of blocking the passage of harmful compounds to the fetus. However, the placenta is not adapted to prevent the passage of man-made compounds that humans have been environmentally and/or occupationally exposed for the past several decades; therefore, fetal exposure to these potentially hazardous compounds cannot be avoided. The placenta is highly sensitive estrogenic activity as it expresses both ERα and ERβ classical estrogen receptors, and, due to its high lipophilicity, environmental EDCs can evade the placental barrier [102]. EDCs have been reported to be transplacental transferred, which indicates that, during embryonic development, the fetus is chronically exposed to mixtures of these compounds, potentially resulting in an early “body burden”, with potentially associated hazardous effects later in live. On the other hand, as discussed above, prenatal exposure to mixtures of xenoestrogens can change epigenetic marks (DNA methylation) patterns in placenta tissues and particular genetic and epidemiological studies demonstrated that parental exposure to EDCs during pregnancy is correlated with decreased birth weight of offspring and shortened anogenital distance in male offspring. Considering the data presented in this work, it is perfectly reasonable to hypothesize that early life EDCs effects may permanently reprogram physiologic processes influencing future health and/or reproductive function [48].

Furthermore, endocrine disruption, mainly associated with estrogenic compounds is also of particular concern for puberty development in males because, during this life stage, accurate endocrine regulation is crucial for the normal development of male secondary sexual characteristics. Currently, it is acknowledged that EDCs can alter the pubertal process by either enhance the advent of puberty (genistein and BPA) or by delay the pubertal onset (Dioxin) [103]. The significance of these findings must be highlighted, as it suggests that EDCs mixtures exposure at sensitive periods of development are likely to cause pronounced detrimental effects on male reproductive system including infertility which is presently a major medical concern.

The results discussed here emphasize the need for further studies to assess additive or synergistic effects of EDC mixtures, particularly during critical and vulnerable life stages and on male reproductive system. Further research must be performed to accurately understand EDCs, principally regarding endocrine systems and mechanistic considerations: (i) normal homeostatic mechanisms (e.g., down-regulation of receptor expression), which may compensate possible effects in adulthood exposures; (ii) exposure during the period of programming of the endocrine system in progress (critical window of exposure) may result in permanent change of function or sensitivity to stimulatory/inhibitory signals; (iii) exposure to the same concentrations of an endocrine signal at different stages in life history may produce different effects; (iv) because of cross talk between different endocrine systems, effects may occur unpredictably; and (v) considerable caution should be exerted in extrapolating in vitro measures of hormonal activity to the situation in vivo [89].

In the context of risk assessment, further considerations should be taken into account, namely the enforcement of biomonitoring of effective doses that could allow the detection of scenarios in which mixture effects may be present, even when the effect would not be predicted from single chemical information, for example with effect-directed fractionation to identify components that should be targets for regulatory attention [3]. However, one of the most important methodological limitations that EDCs mixtures studies face is the fact that, due to the ubiquitous presence of EDCs in the environment and food chain, humans are consistently and chronically exposed to these compounds, which results in an inexistence of negative control groups, i.e., individuals with no contact or exposure to EDCs. These continuous long term exposures in addition to the complexity of short- or long-half-life compounds and bioaccumulation processes still lead to the great challenge of reliably measuring EDCs internal concentrations. The integration of the omics approach combined with state-of-the-art in vitro cell culture models has the potential to considerably improve our understanding of chemical and drug induced perturbations and the replication of reported evidence in independent datasets could provide valid association findings. Nevertheless, considering there are numerous compounds with suspected endocrine disruption potential, to achieve an efficient design, repeated measurements throughout life would have to be performed.

Moreover, it is also important to note that several studies have demonstrated that EDCs effects, particularly at low doses, have associated cell type specificities [104,105]. Thus, cellular effects may vary depending of the utilized in vitro cell systems. The combination of mechanistic toxicity and biokinetics will allow an unparalleled understanding of dynamics and kinetics which can help build prediction models for risk assessment [106] and help to further disclose the unsolved dilemma of synergistic, additive, or antagonistic effects of EDC mixtures. In the next years, educating the public, media, politicians, and governmental agencies on the possible elimination of EDCs from food, water, and air and the creation of products that can test and eliminate potential EDCs are of foremost importance. Additionally, future international collaboration that can assure EDC research funding in basic, clinical, and epidemiological realms is also imperative, especially considering that the cost of research and prevention will result in substantial cost savings in both treatment and mitigation [31].

## 9. Conclusions

Considering the global massive production and consumption of items containing EDCs and consequently the increasing number of individuals exposed to mixtures of these compounds, including pregnant women and young children, where the most damaging impact on later life disease development or abnormal physiology occurs, this overview evidences the urgency of developing and performing valuable and accurate risk assessments to protect exposed individuals and their descendants from the potential hazardous effects of aggregate EDCs mixtures. Importantly, the complexity of EDCs exposure assessments, particular concerning mixture risk assessments, must be carefully considered, and the design of doctrines and data interpretation may require new methodological concepts.

## Figures and Tables

**Table 1:** Effect of EDCs mixtures in human cell lines.

EDCs Mixtures	Concentrations	Cell Lines	Results	References
o,p′-DDT, p,p′-DDT, p,p′-DDE, and β-hexachlorocyclohexane	1–10 nM	MCF7 breast cancer cells	Concentration additive effects	[91]
E2, estrone, BPA, butyl benzylphthalate, endosulfan, methoxychlor, and pentachlorophenol	10–400 nM	MCF7 breast cancer cells	Additive, Antagonistic and synergistic effects	[90]
Benzo[a]pyrene, 1,2-benzanthracene, chrysene, methoxychlor, o,p′-DDT, dieldrin, E2, and genistein	low range (individual chemical thresholds) and a high range (2–10 higher)	MCF7 breast cancer cells	Concentration additivity; antagonistic effects	[92]
17beta-estradiol (E2), ethinyl estradiol, diethylstilbestrol, epidermal growth factor, insulin-like growth factor-I	E2/DES (0–10^−9^ M); EE (0–10^−10^ M); E2 (0–10^−10^ M); EGF/IGF-I (0–10^−9^ M)	MCF7 breast cancer cells	Additive and greater-than-additive interaction	[93]
E2, EE2, genistein, BPA, 4-nonylphenol, and 4-tert-octylphenol	-	MCF7 breast cancer cells	Additive and Antagonistic effects	[94]
2-hydroxy-4-methoxy-benzophenone (BP-3), 2,4-dihydroxy benzophenone (BP-1), octyl methoxy cinnamate (OMC) and 3-(4-methylbenzylidene) camphor (4-MBC)	100 nM–1 µM	MCF7 breast cancer cells	Additive interaction; Estrogen-regulated transcription	[97]
BPA, Butylparaben, Coumestrol, *o,p′*-DDT, DES, Dienestrol, Endosulfan α (I), Endosulfan β (II), 17β-estradiol, Estriol, Estrone, 17α-Ethinylestradiol, genistein, β-HCH, Hexestrol, Kepone, Mestranol, Methoxychlor, Propyl paraben, Zearalenone	10 pM–10 nM	MCF-7 breast cancer cells	Normal and overestimated concentration additivity	[95]
E2, BPA, genistein	GN 1.0, 2.5, 5.0, 7.5 and 10 × 10^−5^ M in the presence of 10^−9^ M of E2 or 10^−5^ M of BPA.	Ovarian cancer cell line BG-1	Suppression of BPA ERα mediated proliferation by GN	[39]
Bitertanol, propiconazole, cypermethrin, terbuthylazine, malathion	10^−10^–10^−5^ M	Human breast carcinoma MVLN cells	ER, AR endocrine-disruption	[34]
Homosalate, nonylparaben, padimate O, benzophenone-3, chlorophenothane, triclosan, 3-(4-methylbenzylidene) camphor, benzal camphor, α-zearalenol, 4-octylphenol, dibutyl phthalate.	0.1, 1, and 10 μM	Sperm cells	Pronounced Ca2+ response.	[98]
BPA, genistein and daidzein.	1 μM	MCF7 and HeLa	Additive effects	[38]
Nonylphenol (NP) and BPA	NP (0–100 μM) BPA (0–5000 μM)	Human Prostate Epithelial Cell Line RWPE-1	Synergistic effects	[96]
BPA, di-ethylhexyl-phthalate (DEHP), dibutyl phthalate (DBP) and 4-tert-octylphenol (4-OP)	0.001–10 μM	Human Macrophage-Like THP-1 Cell	Reduction of phagocytosis; disturbance ER-dependent effects	[99]
Perfluorinated and brominated	10.000, 5000, 1000 and 500 times the serum levels	Human hepatocarcinoma (HepG2) cells	Synergistic effects	[44]
